# Percutaneous ultrasound-guided cryoablation for early-stage primary breast cancer: a follow-up study in Japan

**DOI:** 10.1007/s12282-024-01584-4

**Published:** 2024-04-27

**Authors:** Hisanori Kawamoto, Koichiro Tsugawa, Yuko Furuya, Kaori Sakamaki, Sayoko Kakimoto, Mina Kitajima, Mariko Nagai Takishita, Mizuho Tazo, Mari Hara Nakano, Takako Kuroda, Ayaka Shimo, Arata Shimo, Yasuyuki Kojima, Makiko Tsuzuki, Ai Motoyoshi, Ei Haku, Toru Nishikawa, Yoshihide Kanemaki, Hidefumi Mimura, Mamoru Fukuda

**Affiliations:** 1 Breast and Imaging Center, St. Marianna University School of Medicine, Kawasaki-Shi, Japan; 2 Department of Breast and Endocrine Surgery, St. Marianna University School of Medicine, Kawasaki-Shi, Japan; 3 Department of Diagnostic and Interventional Radiology, St. Marianna University School of Medicine, Kawasaki-Shi, Japan

**Keywords:** Breast cancer, Cryoablation, Cryotherapy, Outpatient procedure, Nonsurgical ablation

## Abstract

**Background:**

Ultrasound-guided percutaneous cryoablation (PCA) for early-stage breast cancer (ESBC) can be performed under local anesthesia in an outpatient clinic. This study continues a pilot stage to examine local control, safety, patient quality of life (QoL), satisfaction and cosmetic outcomes of cryoablation for ESBC.

**Methods:**

PCA was performed under local anesthesia for patients with primary ESBC, followed by radiation and endocrine therapies. Oncologic outcomes were examined by imaging (mammography, ultrasound, MRI) at baseline and 1, 6, 12, 24, 36, and 60 months post-cryoablation. EQ-VAS, EQ-5D-5L, subjective satisfaction and Moiré topography were used to measure health-related QoL outcomes.

**Results:**

Eighteen patients, mean aged 59.0 ± 9.0 years, mean tumor size 9.8 ± 2.3 mm, ER + , PR + (17/18), HER2-, Ki67 < 20% (15/18), underwent PCA and were followed for a mean of 44.3 months. No serious adverse events were reported, and no patients had local recurrence or distant metastasis in the 5-year follow-up. Cosmetic outcomes, satisfaction level, and QoL all improved post-cryoablation. Five-year average reduction rates of the cryolesion long, short, and depth diameters, on US, were 61.3%, 42.3%, and 22.8%, respectively, compared to the 86.2% volume reduction rate on MRI. The correlation coefficient between MRI and US measurement criteria was highest for the long diameter. During follow-up, calcification of the treated area was observed in 13/18 cases.

**Conclusion:**

Cryoablation for ESBC is an effective and safe procedure with excellent cosmetic outcomes and improved QoL. This study contributes to the growing evidence supporting cryoablation as a potential standard treatment for ESBC, given compliance to pre-defined patient selection criteria.

**Supplementary Information:**

The online version contains supplementary material available at 10.1007/s12282-024-01584-4.

## Introduction

Over the past two decades, the widespread adoption of screening mammography and advances in imaging have facilitated the detection of small early-stage breast tumors. This, alongside the successful demonstration of good local tumor control using local therapies, has paved the way to treatment de-escalation, favoring less invasive and nonsurgical resection techniques [1–4]. Minimally invasive nonsurgical techniques aim for curative outcomes while also acknowledging the importance of preserving or enhancing the quality of life (QoL) and the cosmetic appearance following the procedure. The most studied and widely used nonsurgical ablative techniques for the treatment of breast cancer include cryoablation, high-intensity focused ultrasound surgery (HIFU), radiofrequency ablation (RFA), microwave ablation (MWA), and laser ablation (LA) [5, 6].

While breast cancer incidence and mortality are on the decline in many countries, Japan is experiencing an upward trend [7, 8], necessitating more frequent screening and mammography. Combined with improvements in breast cancer imaging and associated with the detection of even microscopic breast cancers that are non-palpable, the demand for nonsurgical treatment is expected to increase. In contrast to United States, where data show a trend toward older age incidence [9], in Japan, breast cancer incidence peaks in the late 40s and early 60s [10–12]. Women in this age group often occupy important societal and family positions. Consequently, there is a growing demand for a treatment option that minimizes hospitalization and recovery time while ensuring favorable clinical and cosmetic outcomes. Cryoablation is a nonsurgical minimally invasive technique using extreme cold temperatures to destroy benign or malignant tumors that meet those requirements. In recent years, it has made rapid progress in clinical studies for early-stage breast cancer, exhibiting local control rates comparable to those achieved in breast-conserving surgery [13–15]. Cryoablation for breast tumors can be performed in an outpatient setting under local anesthesia benefits from the analgesic effect provided by the freezing itself [3, 16] and with satisfactory cosmetic results [17]. Most patients do not have to be hospitalized and can typically resume normal activities after the procedure without significant delay [16].

There are a limited number of studies that have examined long-term ipsilateral breast tumor recurrence (IBTR), cosmetic results, patient satisfaction, and intramammary changes after cryoablation. In this study, a comprehensive 5-year assessment including IBTR rate, complication, long-term cosmetic outcomes, patient satisfaction, health-related quality of life, as well as intramammary changes using MRI and US are presented in continuation to the preceding clinical trial reported in 2021[18].

## Materials and methods

This study was approved by the Ethics Review Board of St. Marianna University (Approval number: No. 4128) and conducted at St. Marianna University Breast & Imaging Center, Kawasaki City, Japan, and informed consent was obtained from all individual participants included in the study.

### Patient population

Included patients met the following criteria (detailed in the pilot study [18]): adult women aged 20 to 85 years with a diagnosis of unifocal invasive ductal carcinoma of the breast (IDC), Eastern Cooperative Oncology Group Performance Status (ECOG) 0 or 1, hormone receptor-positive, Human epidermal growth factor receptor 2 (HER2) negative, Ki-67 positivity ≤ 20%, unifocal primary lesion detectable by mammography (MG), ultrasound (US), or magnetic resonance imaging (MRI), lesion size ≤ 15 mm, negative sentinel lymph node (SLN) biopsy results, and amenable to radiation therapy (RT).

Exclusion criteria included patients with invasive lobular carcinoma, invasive micropapillary carcinoma, intraductal breast lesions, and lesions closer than 5 mm to the skin and pectoralis major muscle. Patients were referred to cryoablation due to their preference or physician recommendation, and those eligible for the study were included.

### Procedure and follow-up

US-guided percutaneous cryoablation was performed under local anesthesia in an outpatient clinic using a liquid nitrogen-based system and a 10G/140 mm cryoprobe (ProSense™ cryoablation system, IceCure Medical Ltd., Israel), as detailed in the pilot study [18]. Briefly, saline hydro-dissection was employed to protect the skin or muscle during freezing when applicable. The ice ball, produced by the cryoprobe during the freeze cycle, was repeatedly measured with US throughout the ablation procedure to ensure full tumor coverage with adequate margins (≥ 10 mm). Wherever necessary, cryoablation cycles were repeated to obtain the necessary size of the ice ball. After the cryoablation procedure, all patients received whole breast irradiation therapy, 50 Gy/25 times, followed by endocrine therapy (ET) (one patient stopped receiving ET one year after cryoablation due to side effects). Imaging (MG, US, and MRI) were used to determine oncologic outcomes during follow-up at 1, 6, 12, 24, 36, and 60 months post-cryoablation. VAB at baseline, at 1 month (prior to RT and ET) or at 6 months (after starting RT and ET) post-cryoablation treatment and imaging (MG, US and MRI) were performed for the first 7 patients [18].

### Tolerance/patient satisfaction/health-related quality of life

Patient tolerance/satisfaction and QoL were recorded at baseline and subsequently assessed 6, 12, 24, 36, and 60 months post-cryoablation procedure. The Moiré topography (MT) assessment (Moiré camera KA-431, Nihon Light Service, Inc., Japan), a non-contact and non-radiological method, was used to analyze the three-dimensional characteristics of the breast surface (e.g., the surface contours and shape of the breast). A 5-point scale was used as a measure of subjective patient satisfaction (set at 3 as baseline). The EuroQol Visual Analog Scale (EQ-VAS) (a patient self-reported assessment of its overall health-related quality of life on a visual analog scale with values between 100 (best imaginable health) to 0 (worst imaginable health)) and the EuroQol-5 Dimensions-5 Levels (EQ-5D-5L) (a patient self-reported assessment of health across five dimensions: mobility, self-care, usual activities, pain/discomfort, and anxiety/depression, with five levels of severity for each dimension, calculated to index scores that range from − 0.59 to 1, where 1 is the best possible health state) scores were used to measure health-related quality of life.

### MRI and US evaluation post-cryoablation procedure

MRI and US were used to measure oncologic outcomes and examine the altered/damaged/degraded tissue and the residual debris resulting from the freezing process during cryoablation (the intended outcome of the cryoablation procedure on the targeted tissue). This observed tissue alteration is denoted as “cryolesion” throughout this study a term also suggested by others [19]. Cryolesion was evaluated by MRI at 1, 6, 12, 24, 36, and 60 months post-cryoablation. Attractive Basic 3D (PixSpace Co., Ltd., Japan) was used to measure and calculate the volume of the cryolesion from the MRI images. The planar length (long diameter, short diameter, and depth) of the cryolesion was also measured from US images.

### Statistical analysis

Descriptive statistic was used for the characteristics related to the patient and cryoablation procedure. Continuous variables were described using median, mean, standard deviation (SD), minimum, maximum, and percentage. Calculations were performed using Microsoft Excel 2003. Correlations were tested using Spearman's rank correlation coefficient.

## Results

A total of 18 patients, all of Japanese origin with a mean age of 59 years (SD 9, range 43–72 years) underwent ultrasound-guided percutaneous cryoablation as the primary treatment for early-stage breast cancer tumors. The mean tumor size on MRI was 9.9 mm (SD 2.3, range 6–14.5 mm), with histopathology type of IDC (17/18) or mucinous carcinoma (1/18). All were estrogen-receptor positive (ER +) and HER2-negative, and 17/18 patients were progesterone-receptor positive (PR +). For all patients, the estimated performance status by the Eastern Cooperative Oncology Group Performance Status Scale was 0; fully active and able to carry on all pre-disease performance without restriction (Table 1).

**Table 1:** Patient characteristics

Characteristics	Value
*N*	18
Age (years)	
Median (range)	60.3 (43–72)
Mean ± SD	59.0 ± 9.0
Tumor characteristics	
Histopathological type	
Invasive ductal carcinoma (IDC)	17
Mucinous carcinoma	1
Receptor status	
ER +	18
PR +	17
HER2-	18
Ki-67	
1–9%	8
10–19%	8
20%	2
Tumor size by MRI (Max. diameter)	
Median mm (range)	9.9 (6–14.5)
Mean mm ± SD	9.8 ± 2.3
Breast composition (by MG)	
Scattered	5
Heterogeneous dense	12
Extremely dense	1
Background parenchymal enhancement (by MRI)	
Minimal	15
Mild	3

*N* number; *SD* standard deviation; *ER* estrogen receptor; *PR* progesterone receptor; *Her2* human epidermal growth factor receptor 2; *MG* mammogram; *MRI* magnetic resonance imaging

Follow-up ranged from 18 to 68 months, with a mean and median of 34.5 (SD 16.2) and 44.3 months, respectively, and 7/18 patients had follow-up longer than 60 months. For all 18 patients, no residual cancer, site of suspected recurrence or distant metastases were detected during the follow-up (by US/MG/MRI and also by VAB for 7 patients) (Table 2).

**Table 2:** Follow-up and outcome

Characteristics	Value
*N*	18
FU time, months	
Median (range)	44.3 (18 − 68)
Mean ± SD	34.5 ± 16.2
Outcome^1^	
Recurrence status^2^	None (0/18)
Distant metastasis status^2^	None (0/18)
Residual cancer^3^	None (0/18)
Serious adverse events	None (0/18)
Adverse events	1^4^/18
Calcification	
Calcification detected	13/18
Without calcification	5/18

^1^Number of cases/total number of cases;

^2^By imaging

^3^By imaging (*n* = 18) and VAB (*n* = 7)

^4^Breast skin redness

*SD* standard deviation

**Fig. 1:**
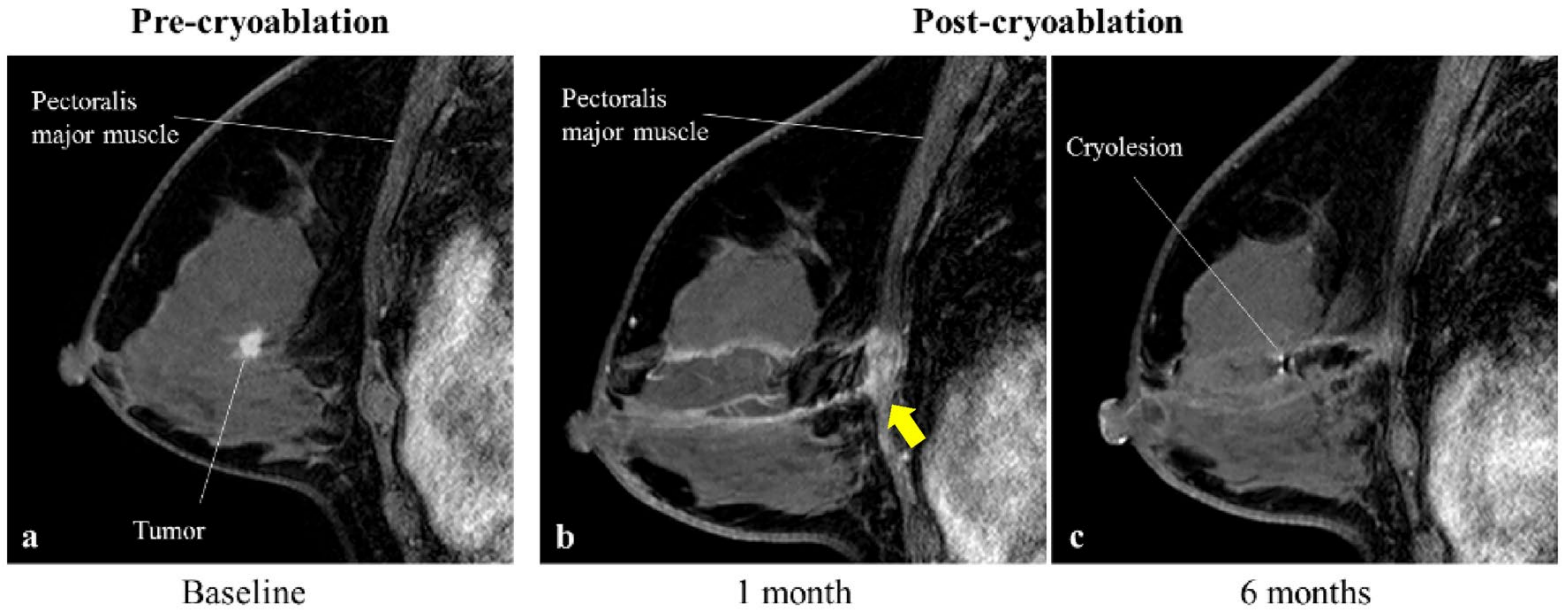
Pre- and post-cryoablation MRI images—pectoralis major muscle burn. MRI images pre- (**a**) 1 month (**b**) and 6 months (**c**) post-cryoablation in a patient 55 years old with 8.9 mm tumor in the left breast. Arrow points to the area of the pectoralis major muscle burns

Only one minor adverse event of skin redness grade 1 (Common Terminology Criteria for Adverse Events (CTCAE) classification) was observed up to one week after cryoablation. This redness resolved spontaneously about 2 weeks after antibiotics and anti-inflammatory and analgesic medications (Table 2). Varying degrees of burns in the pectoralis major muscle were observed by MRI 1 month after cryotherapy in all patients. The burns to the pectoralis major muscle were without symptoms and resolved spontaneously after 6 months (Fig. 1).

### Post-cryoablation MRI imaging and US evaluation

**Fig. 2:**
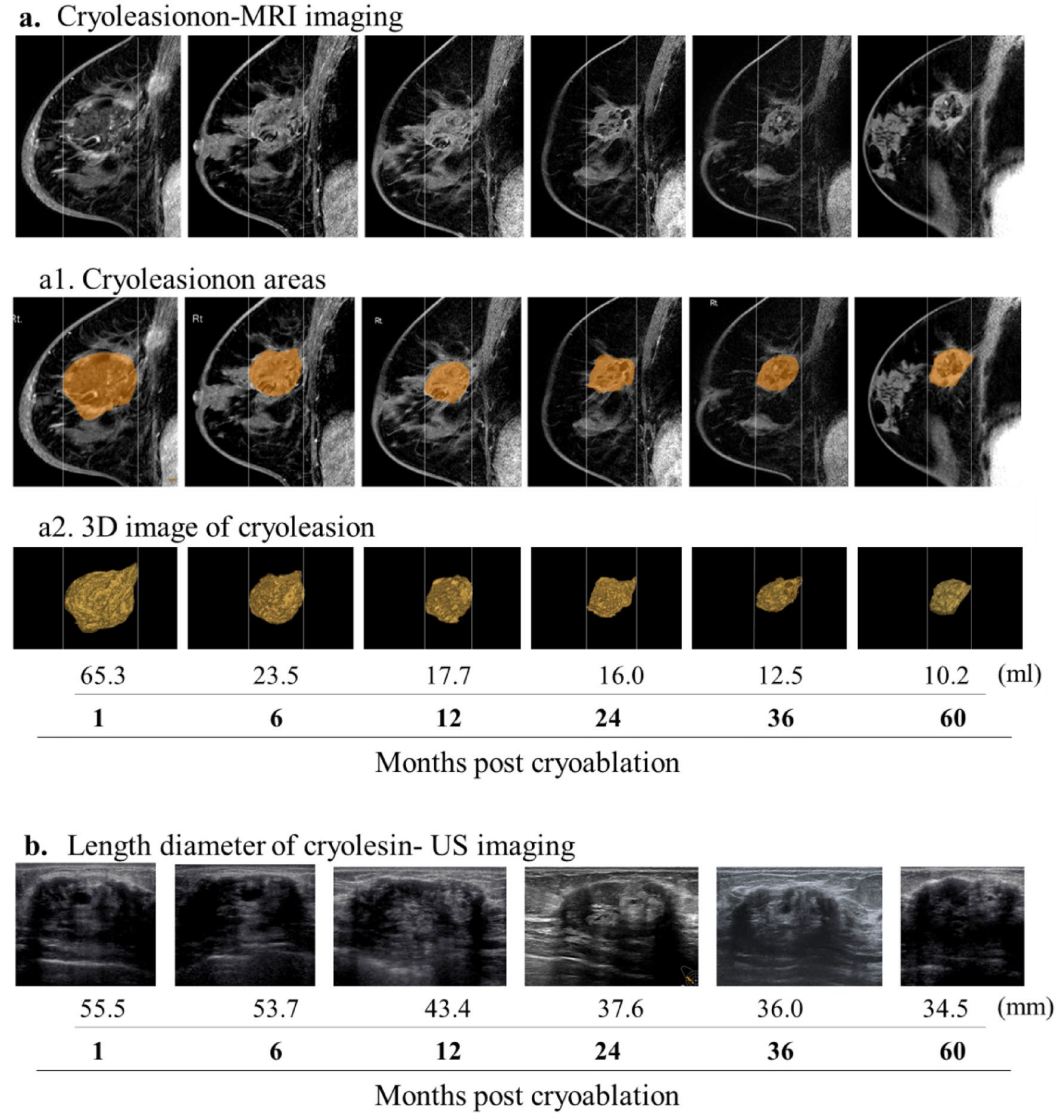
Cryolesion measurements by MRI and US imaging. Post-cryoablation cryolesion images of a patient 67 years old with 12.2 mm tumor in the right breast. Cryolesion’s change over time, as observed by MRI (**a**) were marked (**a1**) and measured, and their volume was calculated (**a2**) using Attractive Basic 3D. Cryolesion as observed by US (**b**)

**Fig. 3:**
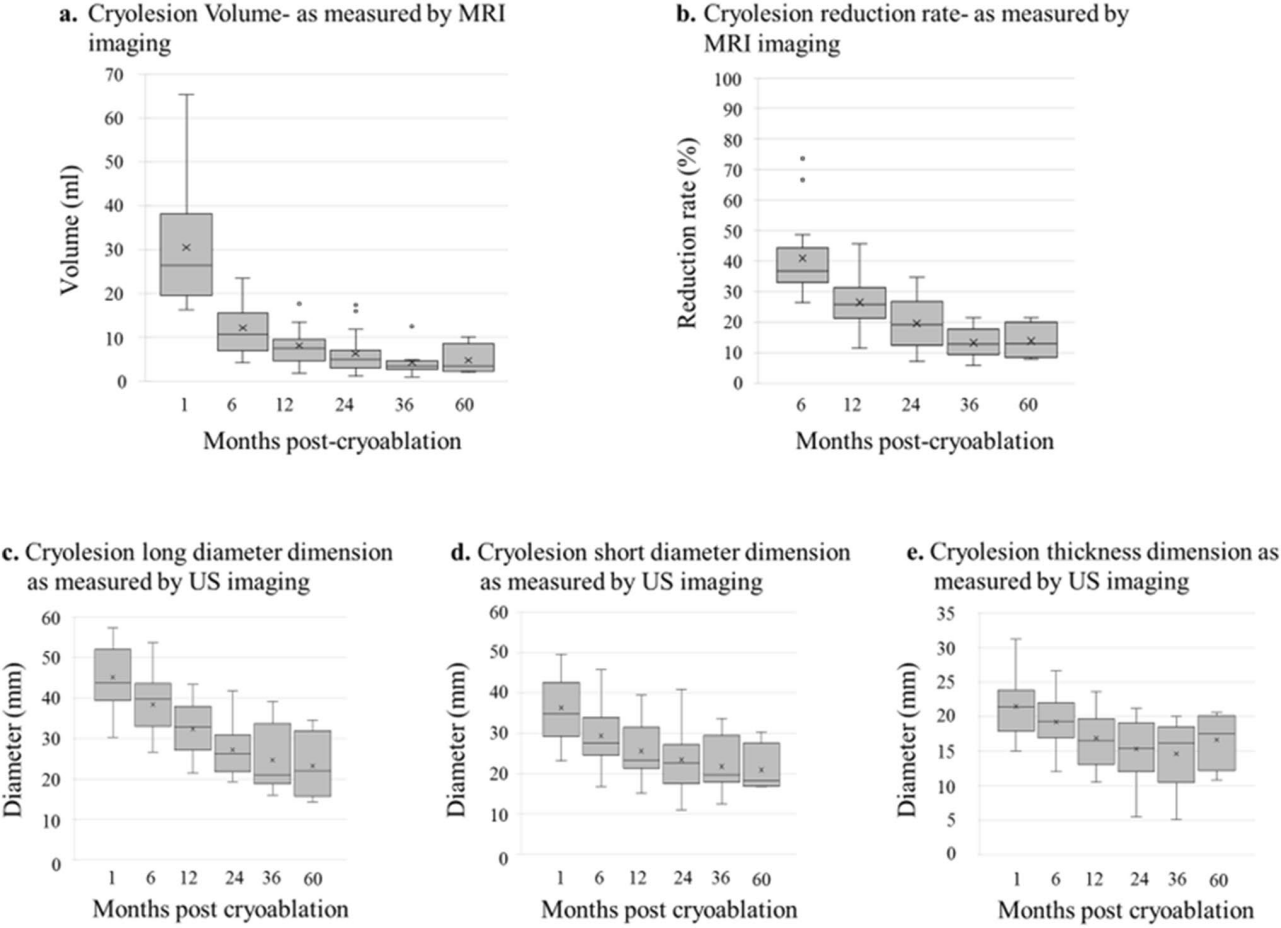
Tissue cryolesion dimension and reduction rates as measured by MRI and US imaging. Post-cryoablation cryolesion volumes (**a**) and reduction rate (**b**), at the indicated post-cryoablation follow-up times, as calculated from MRI images using Attractive Basic 3D. Long (**c**), short (**d**), and depth (**e**) diameter, at the indicated post-cryoablation follow-up times, as measured from US imaging. Mean (x), median (thick line) and outliers (circles) are indicated

The MRI images of the cryolesion at 1, 6, 12, 24, 36, and 60 months were generated using Attractive Basic 3D (PixSpace Co., Ltd., Japan) (Fig. 2a). The mean volume at 1 month was 30.51 ml (range 16.30–65.30 ml), exhibiting considerable variabilities from case to case. This variability was reduced over time (Fig. 3a). Since the cryolesion size occasionally exceeded the tumor size (due to cryoablation ensuring a margin of ≥ 10 mm), volume reduction was calculated based on 1-month measurements rather than baseline. The mean volume reduction rates at 6, 12, 24, 36, and 60 months were 59.06% (range: 26.34–73.62%), 73.48% (range, 54.29–88.55%), 80.4% (range: 65.24–92.77%), 86.74% (range: 78.49–94.11%), and 86.18% (range: 78.49–92.09%) (Fig. 3b).

**Fig. 4:**
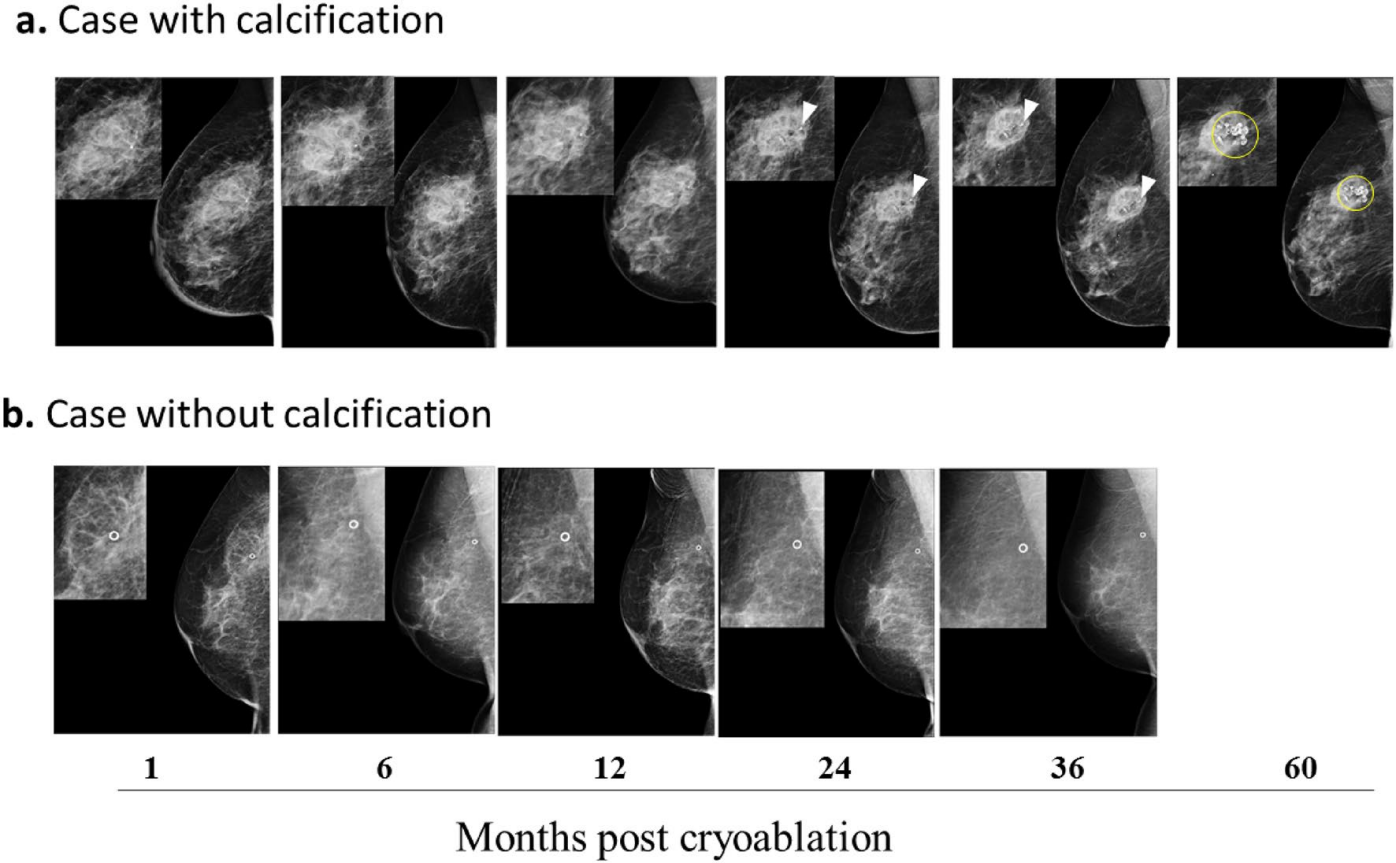
MG image findings of cases with or without calcifications. Patients underwent MG imaging at 1, 6, 12, 24, 36, and 60 months post-cryoablation. Two cases, one of a patient 67 years old with 12.2 mm tumor in the right breast (**a**) and another of a patient 72 years old with 7.7 mm tumor in the right breast (**b**), are presented. Calcification in the spherical area of tissue was observed in some cases after 24 months (**a**), while in others, no calcification was served (**b**). The presence or absence of the calcification was not dependent on any of the tested variables. White arrowheads (**a**) point to the location of the surgical clip. White circles (**b**) are the surgical clips. Yellow circles (**a**) mark coarse calcifications

Cryolesion measurements obtained by MRI were compared with the diameters obtained by US to determine which dimension in the US provides the most accurate representation of the cryolesion’s volume reduction. For this, the long, short, and depth diameters of the cryolesion area at 1, 6, 12, 24, 36 and 60 months (Figs. 3c–e and 4b) were also measured by US. At 60 months, the average reduction of the long diameter, shorter diameter and depth were 61.3%, 42.3%, and 22.8%, respectively (Fig. 3c–e). The correlation coefficient (by Spearman's rank correlation coefficients) for the US long diameter, short diameter, and depth were | *r* |= 0.8085, 0.7976, 0.7752 (all < 0.0001), respectively, with the long diameter exhibiting the highest correlation coefficient.

Throughout the follow-up period, patients underwent MG, revealing the presence of calcification in the spherical area of the cryolesion in 13 patients after 24 months (Table 2 and Fig. 4). The presence or absence of the calcification was found to be independent of various tested variables, including age, the composition of the mammary gland (by MG), background parenchymal enhancement (BPE) (on MRI), or cryolesion volume reduction rate.

#### Cosmetic outcomes/patient satisfaction/health-related quality of life

**Fig. 5:**
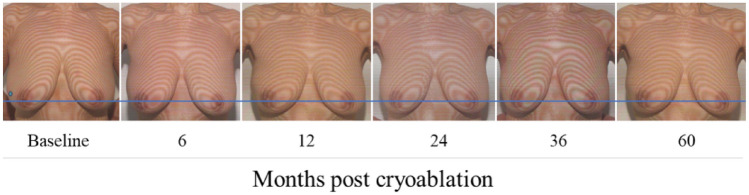
Cosmetic outcomes visualization using Moiré topography. Cosmetic outcomes of a patient 67 years old with 12.2 mm tumor in her right breast were visually assessed using Moiré topography at 6, 12, 24, 36, and 60 months after the cryoablation procedure as compared to baseline. No nipple position distortion, breast deformity, or asymmetry was observed. Blue line—used for alignment

Cosmetic outcomes were visually assessed using Moiré topography at 6, 12, 24, 36, and 60 months after the cryoablation procedure compared to baseline. Cosmetic results were excellent, with no nipple position distortion, breast deformity, or asymmetry observed (Fig. 5).

Subjective patient satisfaction was evaluated on a 5-point scale (with a pre-cryoablation score set at 3 as the reference value). Satisfaction scores consistently surpassed the pre-cryoablation baseline at all time points, with ratings of 4.6, 4.1, 4.5, 4.8, and 4.6 at 6, 12, 24, 36, and 60 months, respectively, highlighting a consistently high level of satisfaction. The EQ-VAS and EQ-5D-5L were used to score the health-related quality of life over time at the same follow-up time as the satisfaction score. No significant change was observed in the patients’ self-reported assessment of overall health-related quality of life by EQ-VAS score (mean of 85.0 and 82.5 at 36 and 60 months, respectively, vs 84.0 at baseline). The EQ-5D-5L index score exhibited a decline from 0.9 before cryoablation to 0.89 at 6 months and 0.87 at 12 months post-cryoablation. Nevertheless, there was an upward trend in scores at 24, 36 and 60 months, reaching 0.94, 0.94 and 0.93, respectively. This trend indicates a comprehensive improvement in patients’ self-reported health assessment across the five dimensions (Supplementary Table S1). When looking at each of the five EQ-5D-5L dimensions, two dimensions, usual-activities and pain/discomfort, showed some decreased scores compared to baseline. The pain/discomfort domain showed the largest reduction, from a baseline median of 2.0 to 1.5 at 12 months and 1.0 at 24 months (Supplementary Table S2).

## Discussion

The present study shows that cryoablation for early-stage low-risk breast cancer is safe, with only one minor complication reported. The results also show that cryoablation is effective, with no local recurrence or metastasis during the 5 years follow-up. Following the evolution of the cryolesion formed post-cryoablation procedure by examining the three-dimensional volumetric measurement MRI and comparing it to the dimensions as measured by US, we demonstrated that the US’ long diameter exhibited the highest correlation coefficient between the two imaging techniques. Both MRI and US image findings showed a significant tumor size reduction rate at 5 years. The study revealed notable enhancements in both patient satisfaction and health-related quality of life, accompanied by excellent cosmetic outcomes when assessing tissue alignment through moiré topography.

In 2021, Fine et al. published the results of a 3-year interim analysis of ipsilateral breast tumor recurrence after cryoablation without excision for low-risk early-stage breast cancer (ICE3 Trial) [20]. ICE3 enrolled 194 patients with an average age of 75 years and a mean tumor size of 8.1 mm. The ICE3 protocol included cryoablation alone with no subsequent resection and long-term follow-up of ipsilateral breast recurrence (IBTR). At the 3-year interim analysis (mean follow-up 34 months), ICE3 was associated with an IBTR rate of 2.06%. Prior to this trial, in 2016, 86 patients (87 treated breast lesions) were included in the ACOSOG phase 2 study to explore the effectiveness of cryoablation in the treatment of invasive breast cancer with tumors smaller than 2 cm. Breast surgery (mastectomy or lumpectomy) was performed within 28 days of cryoablation in the ACOSOG study frame. The results of the trial, reported by Simmons et al. [15] showed a success rate of 75.9% (66/87) for cryoablation. The current study, performed with the same cryoablation system as in Fine et al., showed higher local control rates, although with a smaller sample size.

Currently, there are only a few published studies of imaging findings following breast cancer cryoablation [21, 22] and to the best of our knowledge, none with long-term findings. In the present report, MRI following cryoablation revealed a consistent cryolesion volume reduction for all patients up to 5 years post-procedure. MRI also allowed identifying varying degrees of burns in the pectoralis muscle. The burns were low-grade, without symptoms and with no patients reported related discomfort. Although burns in the pectoralis muscle are acknowledged as a potential complication in cryoablation for breast cancer, their occurrence has not been documented with the same frequency as observed in our study. Burns in the pectoralis muscle is not likely to be detected with other imaging modalities (such as US and MG) and, therefore, may have been missed in other studies. The possibility that the anatomic characteristics of the study population contributed to those findings cannot be ruled out.

MRI is acknowledged as the most accurate method for assessing tumor size/volume compared to MG and US. However, US which remains a widely used imaging modality in various countries, is superior to MG in this regard. By establishing a correlation between the MRI's three-dimensional volume reduction rate and the US's planar length reduction rate, we could determine which parameter on US accurately represents the true volume reduction rate. The results of this study indicate that the long diameter measured on US provides the most accurate reflection of the reduction of the cryolesion volume. Those findings may help others, who use US for follow-up, enabling a more precise assessment of tumor size reduction.

In many patients, coarse calcification was observed 2 years post-cryoablation using MG imaging. The appearance of coarse calcification after cryotherapy for breast cancer has been noted in several other studies [15, 20]. The absence of angiogenesis after tumor freezing is intricately connected to the development of calcification during the tissue's healing process following cryotherapy [23, 24]. Notably, no association was identified between age, mammary gland composition on MG, or the effect of background mammary gland enhancement on MRI to the group with or without calcification.

Together with oncologic control as a measure of effectiveness, it is recognized that other outcomes, such as quality of life, satisfaction, and cosmetic outcome, should be factored into the effectiveness assessment [15, 17, 20]. In the present study, the cosmetics outcomes were excellent, with no bulging, twisting, or deformation of the cryoablation zone. Moiré topography (MT) assessment is a known objective non-radiological and non-contact method that allows for a quick but measurable three-dimensional assessment of body posture while allowing for collecting, storing, and analyzing the obtained data. It is used mainly in Japan and mostly for evaluating spine and/or trunk deviation [25]. In the current study, MT was successfully employed to examine body posture and breast symmetry post-cryoablation, showing excellent results with no nipple position distortion, breast deformity, or asymmetry. While MT has been used post-BC surgery, to the best of our knowledge, it is the first report of using this method post-cryoablation. The successful application of this method suggests its potential as an alternative means to objectively assess and demonstrate cosmetic outcomes following breast cancer cryoablation.

Patient subjective satisfaction was also generally good and comparable to that reported for benign breast fibroadenomas [26]. A notable observation is that, even with a decrease in size after five years, the cryolesion remained palpable for all patients, potentially linked to the anatomical characteristics of the study population Future investigations may include exploring the methods for the early reduction and complete disappearance of cryolesion following cryotherapy.

Criscitiello et al. [27] reported health-related QoL scores for disease-free patients after surgery and adjuvant treatment (either received or under post-adjuvant surveillance) in breast cancer patients. The mean EQ-VAS score in the current study is marginally higher than the one reported in this study but remains within a comparable range (within 1 standard deviation). Also, the mean EQ-5D-5L index score in the present study, 0.90, slightly exceeds the global average of 0.868 and the Japanese average of 0.842 scores reported. Criscitiello et al. [27] also demonstrated that health-related quality of life is higher among patients with early breast cancer. Therefore, the difference in staging between the studies might be a potential factor contributing to the observed disparity in quality of life scores; while the present study reports only on early-stage breast cancer cases, Criscitiello et al. included stages I to III breast cancer patients. Additionally, differences in socioeconomic parameters between the two studies cannot be ruled out. Altogether, the results suggest that the health-related QoL of cryotherapy is equivalent or superior to that of breast-conserving surgery.

While this study presents valuable insights, it is important to acknowledge its limitations, including a small sample size (18 patients) from a single center. Nevertheless, the extended follow-up period and comprehensive outcome measures utilizing various imaging modalities strengthen its importance. Lastly, questions regarding the patients’ socioeconomic status were not included. However, we believe this would not impact the conclusion significantly.

Similarly to cryoablation, radiofrequency ablation is a nonsurgical optional technique used for the treatment of early-stage breast cancer [28]. A large prospective Japanese multicenter clinical trial "Radiofrequency Ablation for Local Therapy of Early Breast Cancer" (RAFAELO study) was initiated in 2013 [29]. 372 patients were enrolled, and the results are awaited. In Japan, RFA will be covered by insurance by December 2023, and introduced into clinical practice. Cryotherapy has shown similar results to RFA in terms of local control rate and tolerability [30–38], and it is expected to be incorporated into clinical practice in the same way as RFA. In the past few years, multiple centers, both domestic and international, have reported excellent local control rates with percutaneous cryoablation for early-stage, low-risk breast cancer tumors smaller than 15 mm from [20–22, 39]. Percutaneous cryoablation has the potential to be an alternative to lumpectomy with promising oncologic results and favorable cosmetic outcomes performed under local anesthesia on an outpatient basis, ensuring a short recovery period for patients.

We have reported not only on the feasibility and safety of percutaneous cryotherapy, but also on its excellent cosmetic outcomes. This study demonstrated the efficacy of the cryoablation technique in eliminating small malignant lesions in the breast, with a promising safety profile. Our study is ongoing with the intent to confirm those results with a larger patient cohort and longer follow-up periods.

### Supplementary Information

Below is the link to the electronic supplementary material.

Supplementary file1 (DOCX 20 KB)

## Data Availability

The datasets generated during and/or analyzed during the current study are not publicly available due to patients’ privacy but are available from the corresponding author on reasonable request.
