# Supplementary material for: Percutaneous ultrasound-guided cryoablation for early-stage primary breast cancer: a follow-up study in Japan
Source: Breast Cancer. 2024 Apr 27;31(4):695–704. doi: 10.1007/s12282-024-01584-4 (PMC11194206; doi:10.1007/s12282-024-01584-4)
Supplement: Supplementary file 1 — Supplementary file1 (DOCX 20 KB) [file 12282_2024_1584_MOESM1_ESM.docx]

## Supplementary material

## Table S1 Patient satisfaction health-related QoL results of EQ-VAS, EQ-5D-5L and subjective satisfaction scales

| **Characteristics** **measures** | | **Months post-cryoablation** | | | | | | |
| --- | --- | --- | --- | --- | --- | --- | --- | --- |
|  |  | **Baseline** | **6** | **12** | **24** | **36** | **60** | |
| **EQ-VAS** | **Median  (range)** | 84.0 (50.0-100.0) | 77.5  (100.0-50.0) | 80.0 (40.0-100.0) | 82.5  (70.0-90.0) | 80.0 (60.0-95.0) | 80.0 (60.0-95.0) | |
|  | **Mean ± SD** | 82.67 ± 13.65 | 75.44 ± 14.55 | 80.88 ± 13.64 | 83.44 ±6.78 | 81.81 ± 10.06 | 81.43 ± 10.25 | |
| **EQ-5D-5L** | **Median  (range)** | 0.90 (0.80-1.00) | 0.90 (0.73-1.00) | 0.89 (0.41-1.00) | 0.95 (0.82-1.00) | 0.90 (0.82-1.00) | 1.00(0.83-1.00) | |
|  | **Mean ± SD** | 0.90 ± 0.07 | 0.89 ± 0.09 | 0.87 ± 0.13 | 0.94 ± 0.06 | 0.92 ± 0.06 | 0.94 ± 0.07 | |
| **Subjective satisfaction** | **Median  (range)** | 3.00 (3.00-3.00) | 5.00 (3.00-5.00) | 4.00 (1.00-5.00) | 5.00 (3.00-5.00) | 5.00 (4.00-5.00) | 5.00 (4.00-5.00) | |
|  | **Mean ± SD** | 3.00 ± 0.00 | 4.64 ± 0.57 | 4.06 ± 1.08 | 4.47 ± 0.78 | 4.82 ± 0.39 | 4.50 ± 0.71 | |
| SD-Standard Deviation | | | | | | | |  |

## Table S2 Patient satisfaction health-related QoL results of EQ-5D-5L five dimensions

| **Dimensions** | **Parameter** | **Months post-cryoablation** | | | | | |
| --- | --- | --- | --- | --- | --- | --- | --- |
|  |  | **Baseline** | **6** | **12** | **24** | **36** | **60** |
| **Mobility** | **Median**  **(range)** | 1.00  (1-2) | 1.00  (1-2) | 1.00  (1-4) | 1.00  (1-1) | 1.00  (1-2) | 1.00  (1-2) |
|  | **Mean ± SD** | 1.06 ± 0.23 | 1.11± 0.31 | 1.28 ± 0.73 | 1.00 ± 0.00 | 1.13 ± 0.33 | 1.14 ± 0.35 |
| **Self-care** | **Median**  **(range)** | 1.00  (1-2) | 1.00  (1-1) | 1.00  (1-1) | 1.00  (1-1) | 1.00  (1-1) | 1.00  (1-1) |
|  | **Mean ± SD** | 1.06 ± 0.23 | 1.00 ± 0.00 | 1.00 ± 0.00 | 1.00 ± 0.00 | 1.00 ± 0.00 | 1.00 ± 0.00 |
| **Usual activities** | **Median**  **(range)** | 1.00  (1-2) | 1.00  (1-3) | 1.00  (1-4) | 1.00  (1-2) | 1.00  (1-1) | 1.00  (1-1) |
|  | **Mean ± SD** | 1.17 ± 0.37 | 1.17 ± 0.50 | 1.39 ± 0.76 | 1.06 ± 0.24 | 1.00 ± 0.00 | 1.00 ± 0.00 |
| **Pain/ Discomfort** | **Median**  **(range)** | 2.00  (1-3) | 2.00  (1-3) | 1.50  (1-4) | 1.00  (1-2) | 1.00  (1-2) | 1.00  (1-2) |
|  | **Mean ± SD** | 1.83 ± 0.69 | 1.67 ± 0.58 | 1.72 ± 0.93 | 1.44 ± 0.50 | 1.38 ± 0.48 | 1.29 ± 0.45 |
| **Anxiety /Depression** | **Median**  **(range)** | 1.00  (1-2) | 1.00  (1-4) | 1.00  (1-2) | 1.00  (1-2) | 1.00  (1-2) | 1.00  (1-2) |
|  | **Mean ± SD** | 1.11 ± 0.31 | 1.39 ± 0.76 | 1.22 ± 0.42 | 1.06 ± 0.24 | 1.13 ± 0.33 | 1.14 ± 0.35 |
